# Electronic and optical properties of topological semimetal Cd_3_As_2_

**DOI:** 10.1038/srep45500

**Published:** 2017-04-06

**Authors:** Adriano Mosca Conte, Olivia Pulci, Friedhelm Bechstedt

**Affiliations:** 1 ISMN, Consiglio Nazionale delle Ricerche, Via Salaria Km 29,300 00015 Monterotondo Stazione (Roma) - Italy; 2 Dipartimento di Fisica, Università di Roma ‘Tor Vergata’, Via della Ricerca Scientifica 1, I-00133, Rome, Italy; 3 I.N.F.N, - Sezione di Roma ‘Tor Vergata’, and CNR-ISM, Roma, Italy; 4 IFTO, Friedrich-Schiller-Universität and ETSF, Max-Wien-Platz 1, 07743 Jena, Germany

## Abstract

Using *ab initio* density functional theory the band structure and the dielectric function of a bct Cd_3_As_2_ crystal are calculated. We find a Dirac semimetal with two Dirac nodes **k**_±_ near the Γ point on the tetragonal axis. The bands near the Fermi level exhibit a linear behavior. The resulting Dirac cones are anisotropic and the electron-hole symmetry is destroyed along the tetragonal axis. Along this axis the symmetry-protected band linearity only exists in a small energy interval. The Dirac cones seemingly found by ARPES in a wider energy range are interpreted in terms of pseudo-linear bands. The behavior as 3D graphene-like material is traced back to As *p* orbital pointing to Cd vacancies, in directions which vary throughout the unit cell. Because of the Dirac nodes the dielectric functions (imaginary part) show a plateau for vanishing frequencies whose finite value is proportional to the Sommerfeld fine structure constant but varies with the light polarization. The consequences of the anisotropy of the Dirac cones are highlighted for the polarization dependence of the infrared optical conductivity.

Topology–dependent electronic properties of solids are subject of considerable current interest^1^,^2^. In particular, the appearance of ultrarelativistic particles in Dirac cones opens access to novel physics in topological matter. Topological insulators (TIs) are a new class of such materials^3^,^4^. Their surfaces or interfaces as well as edges exhibit topologically protected two-dimensional (2D) or one-dimensional linear bands in their fundamental gap as first demonstrated for heterojunctions of the band-gap-inverted HgTe zinc-blende crystals^5^,^6^. Another exotic material class is built by topological semimetals, in particular Weyl semimetals (WSMs)^1^,^2^ sometimes, in the three-dimensional (3D) case with higher symmetry, called 3D Dirac semimetals^7^. Unlike two-dimensional (2D) TIs, which represent a quantum spin Hall phase described by a single topological invariant Z_2_, a 3D TI is characterized by four invariants^8^. A WSM is characterized by the monopole-antimonopole separations in momentum space^2^. A WSM is a new topological state of 3D quantum matter with Weyl nodes at the Fermi level in the bulk and Fermi arcs on the surface^9^. Around Weyl nodes the low-energy physics is given as 3D two-component Weyl fermions^10^. To get a WSM, either time-reversal (T) or inversion (I) symmetry should be broken. In the case of both T and I symmetries, one expects a 3D Dirac semimetal described as four-component Dirac spinors^11^ with linear dispersion, which can be considered as two distinct Weyl fermions with opposite chirality. The band touching point is the Dirac point but sometimes still called Weyl point. The presence of Fermi arcs on a surface between two corresponding Weyl points whithin the WSM (or even a Dirac semimetal)^9^ has been recently predicted theoretically^12^,^13^ and confirmed experimentally by means of angle-resolved photoemission spectroscopy (ARPES) for the compounds Na_3_Bi and Cd_3_As_2_^14^,^15^,^16^. The ultra-high carrier mobility measured for Cd_3_As_2_^17^ supports the existence of linear bands and massless Dirac or Weyl fermions. High-quality needle-like or large chunky Cd_3_As_2_ crystals can be grown using excess Cd^17^. Indeed, the existence of stable Cd_3_As_2_ solids has been known for years^18^,^19^. Of particular interest were their semimetallic character and high electron mobility^20^. The electronic structure of Cd_3_As_2_ has been calculated^13^ for the primitive tetragonal (pt) polymorph with space group P4_2_/nmc 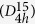 and 40 atoms in the primitive unit cell, which should appear at intermediate temperatures (475 °C)^18^. Calculations have been performed also for the non-centrosymmetric, as well as body-centered tetragonal (bct) structure with I4_1_/cd 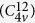 space group and 80 atoms in the unit cell, proposed for temperatures below 475 °C^19^. The basic geometry is given as an anti-fluorite arrangement of the atoms with one fourth Cd site vacancy (see Fig. 1). With the corresponding 10-atom building block the pt and bct structures can be constructed. The pt structure with lattice constants *a* = 8.95 Å and *c* = 12.65 Å has As atoms in an fcc array and six Cd ones in fluorite-like positions in a 10-atom subsystem. The bct phase has a larger unit cell with *a* = 12.67 Å and *c* = 25.48 Å^21^ in which the empty cube Cd vertices are ordered in 3D rather than in a 2D array.

A very recent X-ray reinvestigation of the bct polymorph^21^ found a centrosymmetric rather than a non-centrosymmetric symmetry with a distorted superstructure of the antifluorite type with lattice constants *a* = 12.633 Å and *c* = 25.427 Å and space group I4_1_/acd 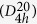. These findings are important for the topological character of Cd_3_As_2_. The presence or absence of inversion symmetry has implications on its band structure. In the non-centrosymmetric case the spin degeneracy of the bands in the vicinity of a Weyl point is lifted and Cd_3_As_2_ is an example for a Weyl semimetal^13^. With inversion symmetry the compound represents a 3D Dirac semimetal with no spin splitting. This makes Cd_3_As_2_ a 3D electronic analogue of graphene^21^. This relationship has however not been proven by an orbital analysis. The appearance of linear bands is claimed to be found in the ARPES measurements^14^,^15^,^16^. Other electronic structure studies, e.g. indirect ones by means of optical spectroscopies, became available just recently^22^,^23^.

In this paper we study the influence of the stacking of basic units of Cd_3_As_2_ on the electronic structure and optical properties at vanishing frequencies. We investigate the analogy of a Dirac semimetal to graphene. For that purpose, we relate the 3D Dirac cones to the topological 3D arrangements of As *p* orbitals with varying orientation. We distinguish between the Dirac electrons near the Fermi energy and Kane electrons away from it by about 1 eV. The influence of the character as topological semimetal on the optical properties, especially the optical absorption, is a special topic of the paper. In particular, the influence of linear bands near Dirac points on the frequency dependence of the optical conductivity is discussed. The anisotropy of the Dirac cones governs its polarization dependence.

## Results

### Cd_3_As_2_ building block: simple cubic substructure

**Figure 1:**
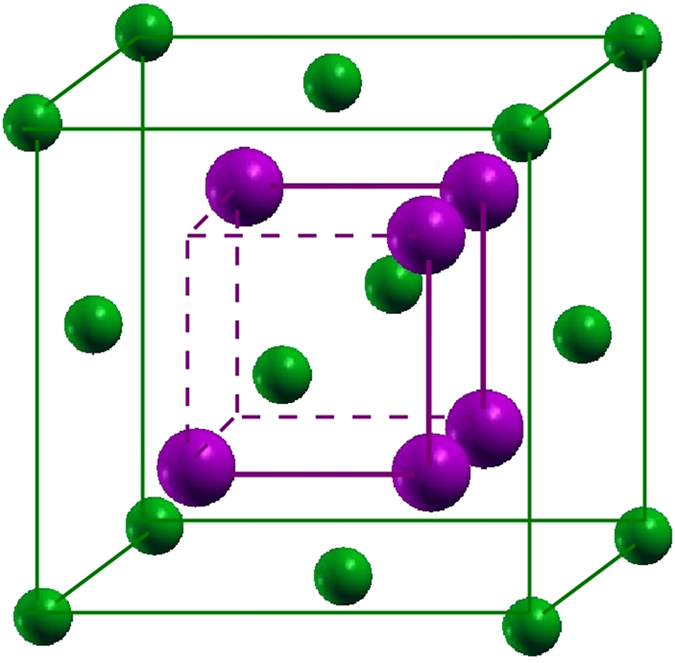
Basic geometry of Cd_3_As_2_ represented by a 10-atom cube which is a building block for the 80-atom bct structure. It is made of an fcc sublattice of As atoms (green) each surrounded by 6 Cd atoms (purple) disposed at cube vertexes as in an anti-fluorite arrangement but with two Cd vacancies at diagonal corner points of one face of the cube.

**Figure 2:**
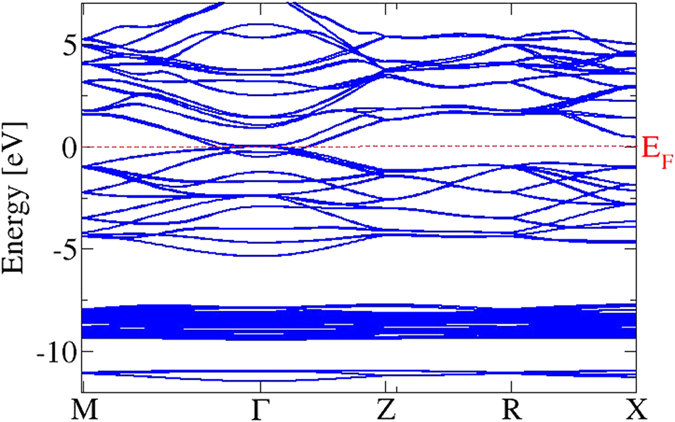
Electronic band structure of the 10-atom substructure of Cd_3_As_2_ with SOI in a wide energy range. The Fermi energy is used as energy zero.

**Figure 3:**
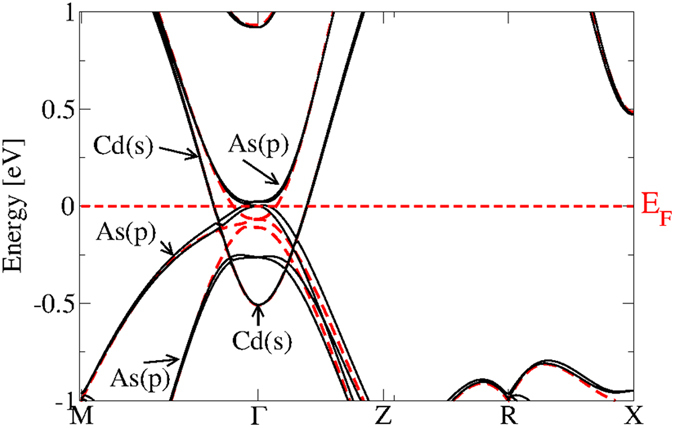
Electronic band structure of the 10-atom substructure of Cd_3_As_2_ with (black) and without (dashed red) SOI. The orbital character of the corresponding Bloch states is reported.

Several basic properties of Cd_3_As_2_ polymorphs can be understood within a simplified crystal structure. An fcc sublattice of As atoms with a lattice constant *a*_0_ describes the unit cells (see Fig. 1) of such a model crystal. Each As atom is surrounded by 6 Cd atoms at the corners of a cube with an edge *a*_0_/2. Two Cd vacancies appear diagonally opposite each other in one face of the small cube. As illustrated in Fig. 1, a distorted anti-fluorite structure with two formula units Cd_3_As_2_ and two missing cations appear in a cube of edge length *a*_0_. The relaxed Cd positions are described elsewhere^21^. The resulting lattice constant of the cube is *a*_0_ = 6.46 Å. It indicates that the bct crystals with space groups *I*4_1_/*cd* or *I*4_1_/*acd* may be described by non-primitive tetragonal unit cells consisting of 2 × 2 × 4 = 16 cubes with 160 atoms and hence by primitive bct cells with 80 atoms. We study one cube, that is, one building block of the true bct stucture. The studied model crystal with a simple cubic Bravais lattice is a metal, as can be seen in the band structure in Figs 2 and 3. The bands are plotted versus high-symmetry lines in a simple cubic BZ (see ref. 24) because of the sc translational symmetry. The crystal symmetry is however lower. Due to the Cd vacancies the crystal possesses a low symmetry with a point group only with a rotation by 180 degrees around the rotational axis (−1, 0, 1). The overall band structure in Fig. 2 is rather similar to those of a fluorite crystal, such as CaF_2_, or anti-fluorite one, such as Na_2_O. However, there are distinct features in the details.

Despite the overlap around Γ, the bands in Fig. 2 can be arranged in four groups. The conduction bands have a strong Cd5*s* character in their low-energy parts, as shown in Fig. 3. They are followed by the upper valence bands with strong As4*p* character. Below (around −8 eV) one finds Cd4*d*-derived as well as As4*s*-dominated bands.

The orbital character of the band states near the Fermi level has been obtained by a Löwdin charge population analysis^25^ in Fig. 3. In this energy region around Γ the importance of the SOI is clearly visible. Together with the scalar-relativistic effects it tends to bring the Cd *s* atomic level below the As *p* one leading to a band inversion between As4*p* and Cd5*s*-derived bands at Γ. The situation is similar to the band inversion in the case of the zero-gap semiconductor HgTe crystallizing in zinc-blende geometry^26^. The fourfold degenerate and, hence, the upper spin-orbit-split As *p* level pins the Fermi level. However, the lower symmetry of the Cd_3_As_2_ model crystal enforces its metallicity. The Cd5*s*-derived, low-lying parabolic band crossed by the Fermi energy describes a metal. The band inversion due to the scalar relativistic effects already gives a hint for the non-trivial topological character of Cd_3_As_2_ similar to the cases of the HgTe and *α*-Sn^26^. Another interesting effect of the SOI is the spin splitting of the *p*-like bands away from Γ in agreement with the fact that the studied system is without inversion symmetry. The band structure in Fig. 3 also shows the absence of linear bands crossing near the Fermi level and, hence, the absence of 3D Dirac cones. This fact already shines light on a possible character of a Weyl or Dirac semimetal in the bct geometries with 80-atom unit cells.

**Figure 4:**
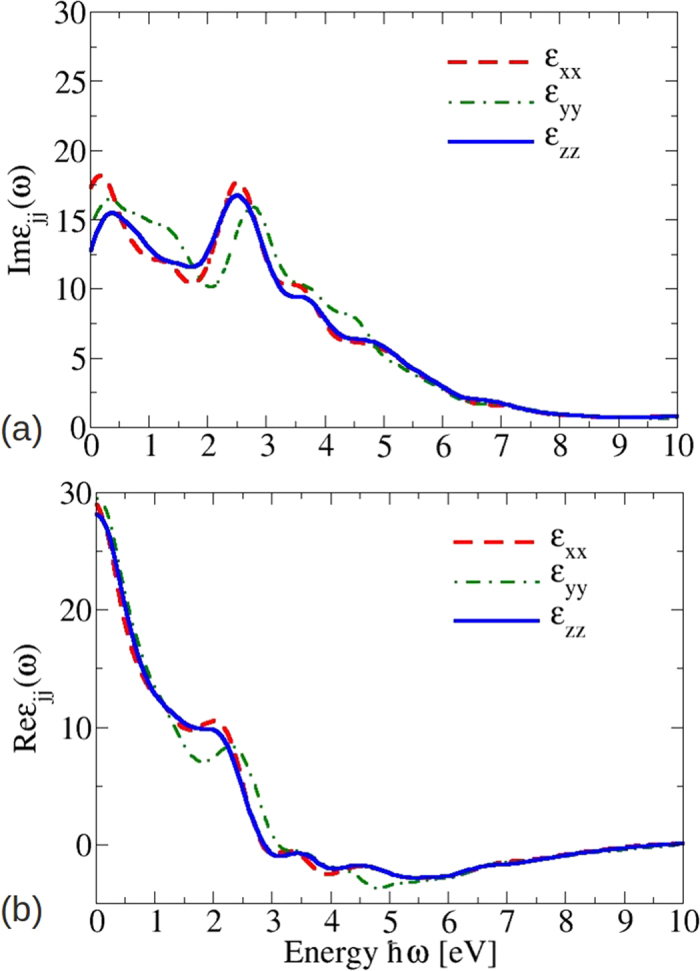
Imaginary (**a**) and real (**b**) part of the dielectric function of the 10-atom substructure of Cd_3_As_2_. The components *xx, yy*, and *zz* are indicated by red, green, and blue lines, respectively. The Cartesian coordinates are identified with the cubic axes.

The consequences of the band structure with SOI on optical spectra are illustrated in Fig. 4. The imaginary part of the interband dielectric function (eq. (8) in Method part) in Fig. 4a clearly illustrates the influence of the band structure. At photon energies of *ħω* ~ 2.6 eV there is a peak mainly due to dipole-allowed optical transitions at the BZ boundary near M points (see Fig. 2). For *ω* → 0 the imaginary parts show the typical behavior for interband transitions with vanishing gap, while the real parts in Fig. 4b diverge in agreement with the metallic character of the artificial material.

### Body Centered Tetragonal Cd_3_As_2_ crystal

The starting atomic positions in a real bct Cd_3_As_2_ crystal have been determined from those in the artificial sc crystal with cubes of edge length *a*_0_ as building blocks. In such a cube four As atoms occupy corner and mid-face positions (0, 0, 0) (0, 1/2, 1/2) (1/2, 0, 1/2) and (1/2, 1/2, 0) of the fcc sublattices. The six Cd atoms surround the As atom in position (0, 0, 0) and sit at the corners of a small cube with edge length *a*_0_/2, thereby letting two positions empty to simulate the vacancies. The fcc sublattice of As atoms is then used to build up a non-primitive tetragonal lattice with Bravais vectors (2, 0, 0), (0, 2, 0) and (0, 0, 4) in units of *a*_0_. The other Cd atom coordinates are instead found by fixing the center of symmetry in the center of the tetragonal cell and applying the symmetry operations of the I4_1_/acd space group to each of the six Cd atoms surrounding the As atom in (0, 0, 0) position. In this way, all the As atoms are surrounded by six Cd-atom cubes with an arrangement of the vacancies that helix inside the bct unit cell as displayed in Fig. 3 of ref. 21. We test this structure applying symmetry operations of the I4_1_/acd space group (see ref. 27). The resulting structure is indeed centrosymmetric. Finally, the tetragonal crystal with 160 atoms in the non-primitive unit cell is reduced to a bct crystal with 80 atoms in the atomic basis and Bravais lattice vectors (200) (020) and (112) in units of *a*_0_. In a final optimization step the atomic positions are allowed to relax until the DFT total energy reaches a minimum. The resulting tetragonal lattice constants amount to *c* = 25.84 Å and *a* = 12.93 Å close to the experimental values^21^.

**Figure 5:**
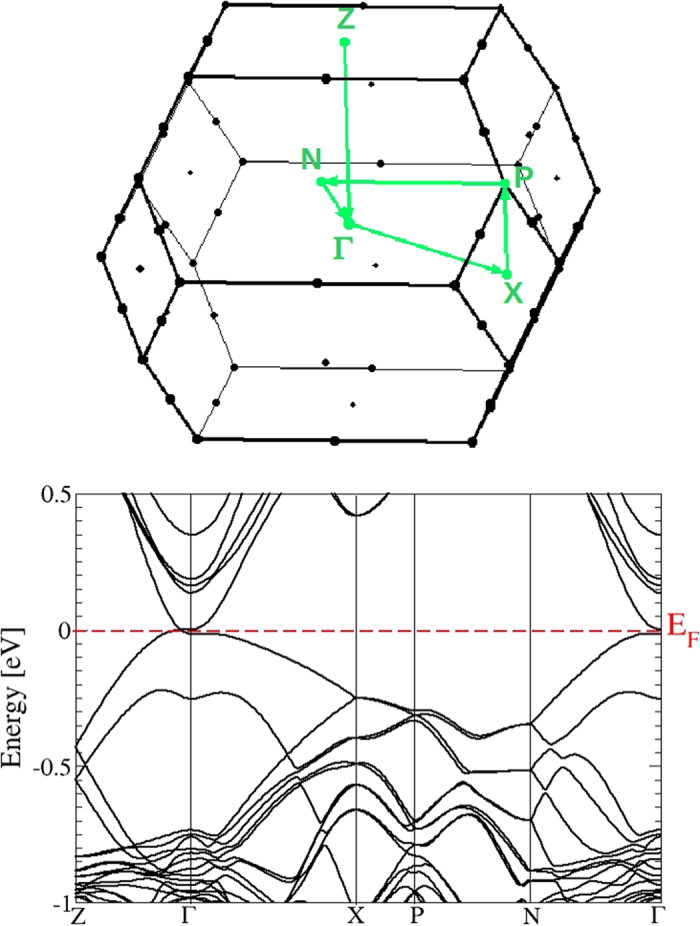
Electronic band structure of the bct Cd_3_As_2_ superstructure with SOI. (**a**) bct BZ with indication of high-symmetry lines and dots. (**b**) Bands versus high-symmetry lines. The Fermi energy is used as energy zero.

The Cd_3_As_2_ band structure calculated with SOI for the relaxed geometry is plotted in Fig. 5 versus high-symmetry lines in the bct BZ. No spin splitting of the bands is visible because of the presence of inversion symmetry. The absolute positions of the band energies, the band dispersions, and the band splittings on the BZ boundary along XP and PN are very similar to those calculated by means of the experimentally determined centrosymmetric geometry and an all-electron DFT approach^21^. All bands are at least twofold degenerate. However, also the non-centrosymmetric bct crystal and a different DFT approach based on projector-augmented waves give very similar results^13^, because of the rather small spin splittings.

**Figure 6:**
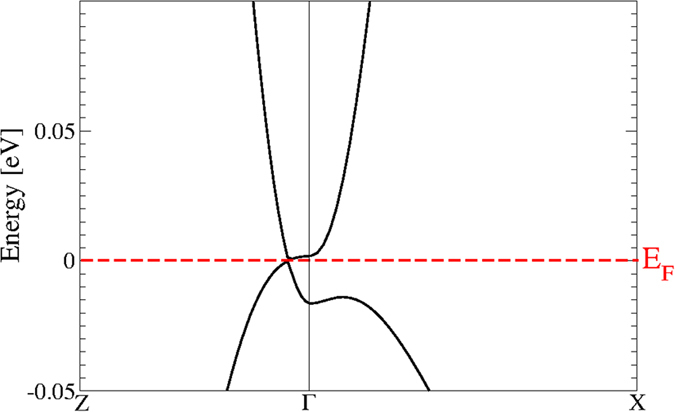
Zoom of the band structure of bct Cd_3_As_2_ near one Dirac point on the tetragonal axis ΓZ with SOI.

**Figure 7:**
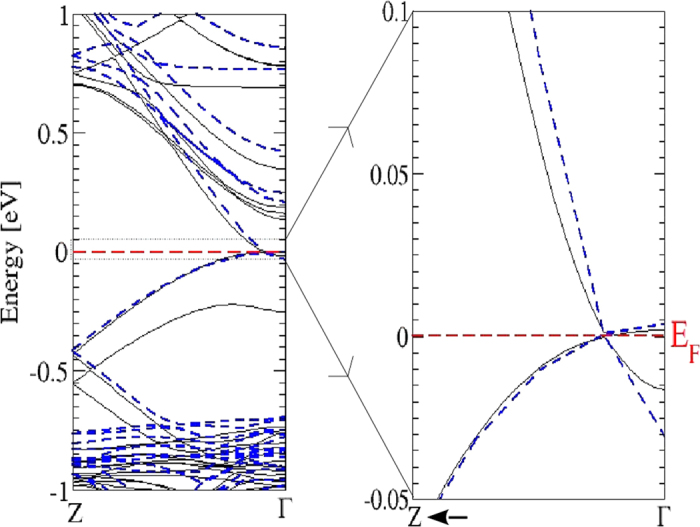
(**a**) Electronic band structures of bct Cd_3_As_2_ along the ΓZ line with (solid black lines) and without SOI (dashed blue lines) (**b**) Zoom for significantly reduced energies.

Most interesting is the behavior of the band structure near the Fermi energy *E*_*F*_ and the BZ center Γ in Fig. 6. Far away from Γ there are no allowed band states in the low-energy region. Despite the band folding effect due to the larger unit cell, the bands around Γ in Fig. 6 show seemingly some similarities to those in Fig. 3. There are As *p* bands crossing the Fermi level. However there are also differences: the Cd *s*-derived band crossing the Fermi level in the small cube here disappeared. As more clearly visible in Fig. 7 for an extremely small energy interval, around Γ a small gap of about 0.02 eV is opened. However, along the ΓZ line (and consequently along the opposite Γ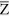 line) twofold degenerate linear bands cross at 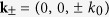, thereby forming two fourfold-degenerate Dirac points at the Fermi level. Therefore the centrosymmetric bct Cd_3_As_2_ forms a topological Dirac semimetal with two Dirac points on the tetragonal axis in a distance ±*k*_0_ from Γ. Similar to graphene, ideal bct Cd_3_As_2_ may be also identified as a multivalley zero-gap semiconductor. In the non-centrosymmetric case^13^,^15^ small band splittings occur and hence Weyl points arise. Around Γ and *E*_*F*_ the band structures in Figs 6 and 7 are in agreement with symmetry considerations^28^. The inverted band structure, already found for the building blocks consisting of two formula units Cd_3_As_2_, cannot open up an energy gap due to the C_4_ rotational symmetry around the tetragonal axis. It protects two 3D Dirac cones touching at two special points **k**_±_ along the ΓZ line^13^. Actually, the linearity of the bands at the Dirac points holds just in a very small energy range, as shown in Fig. 6. Therefore, Dirac fermions only appear for excitation energies much lower as claimed discussing ARPES measurements (see discussion below).

The symmetry protection of the vanishing gap at the Dirac nodes even in the presence of SOI is illustrated in Fig. 7 for bands along the tetragonal axis. This figure enlights the SOI influence. Whereas higher-lying conduction bands and lower-lying valence bands are generally shifted toward higher energies, the bands forming the Dirac cones are hardly influenced along the Γ*Z* direction. The Dirac points are protected by the crystal symmetry (see also arguments in ref. 28). However, also the gap at Γ between the energetically adjacent conduction and valence bands is also only less shrinked by SOI. The relatively small influence of SOI is in agreement with the above results on the artificial sc Cd_3_As_2_ crystal.

**Figure 8:**
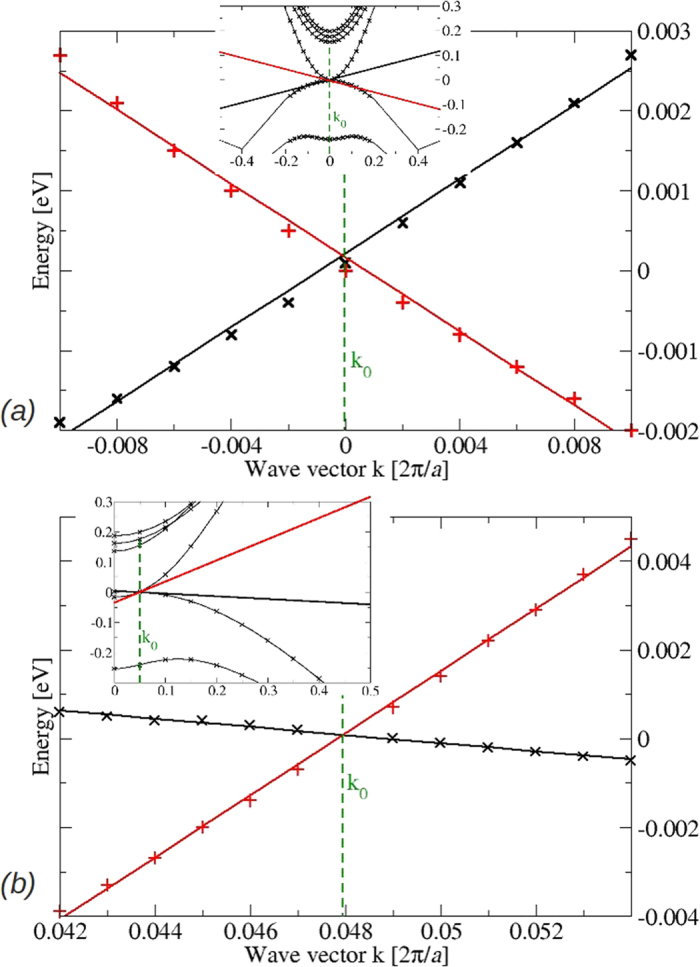
Empty and occupied bands (crosses) around the Dirac point 
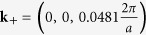
 at ΓZ versus wavevector **k**_‖_ in the *xy*-plane (**a**) and 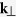 along the *z* coordinate (**b**). The linear fits together with the resulting three Fermi velocities 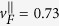, 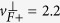, and 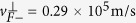 to characterize the Dirac cones (1). The insets display the stronger band dispersion around Dirac node if larger energy and wavevector intervals are studied.

The details of the empty (+) and filled (−) band around a Weyl or Dirac point can be described by the hyberbolic dispersion formula (for a node on the positive ΓZ line)


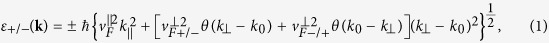


where *k*_‖_ lies in the *xy* plane and *k*_⊥_ is along the tetragonal axis *z*. The corresponding linear fits around *k*_⊥_ = *k*_0_ and *k*_‖_ = 0 for a small energy interval are depicted in Fig. 8a,b. The three values of the Fermi velocity, resulting in units of 10^5^ m/s are 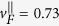, 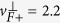, and 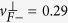, indicate the symmetry of the Dirac cones in the *xy*-plane but the considerable asymmetry along the tetragonal *z* axis. The positions of the two Dirac nodes in **k** space are given by *k*_0_ = 0.0481 2*π/a* = 0.099 Å^−1^ using the optimized lattice constant *a* = 12.93 Å. From the insets of Fig. 8 it is clear that the linearity of the bands at the Fermi level holds just for an extremely small range of energies and just very near the Dirac points.

The computed values differ significantly from the values *k*_0_ = 0.032 Å^−1^ and *v*_*F*_ = 3 × 10^5^ m/s derived for isotropic Dirac cones^13^ or *k*_0_ = 0.12 Å^−1^, 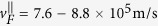, and 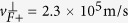
29. Optical measurements^22^ indicate energy-dependent average Fermi velocities which range from 1.2 × 10^5^ to 3 × 10^5^ m/s somewhat larger than our computed values. The comparison with ARPES data^15^,^16^,^29^ is difficult because of the different surface orientations, the study of relatively large energy intervals, where the linear bands presented in Fig. 8 change their dispersion into a pseudolinear behavior (see inset), and the necessary refolding of measured bands around the center of the surface BZ to bands around the Dirac points with finite *k*_0_ value. Therefore, the Fermi velocities derived from ARPES are hardly compatible with the ones derived from Fig. 8. This especially holds for the measurement of occupied linear bands up to 5 eV below the Fermi level^29^. Correspondingly, the experimentally derived values of 10 × 10^5^ m/s perpendicular to the surface normal and 3.3 × 10^5^ m/s along the normal of the cleavage face are larger than our theoretical ones. The deviations are larger than the 15–20% quasiparticle increases expected from graphene and other honeycomb sheet materials^30^,^31^. This is illustrated by the insets in Fig. 8. Away from the Dirac points, the band dispersion and, hence, in the linear approximation, the Fermi energies are larger than the fit values for small energies and wave vectors. According to our findings in Figs 6, 7 and 8, symmetry-protected asymmetric Dirac cones only appear at small electron and hole energies, in contrast to ARPES studies^14^,^15^,^16^ but in line with results of scanning tunneling spectroscopy (STS)^32^. The pseudo-linearity observed in ARPES for higher energies may be interpreted in terms of the standard Kane model for semiconductors (see ref. 33), here however for a zero-gap semiconductor^23^. Especially for surfaces not perpendicular to the tetragonal axis, we expect indications for the band asymmetry when approaching one Dirac node from its left or right side along the ΓZ direction in future high-resolution ARPES measurements for extremely low electron energies. The interpretation of the current ARPES studies^14^,^15^,^16^, in which the observed conical feature extending over a wide energy range was identified as symmetry protected Dirac particles, is questionable.

**Figure 9:**
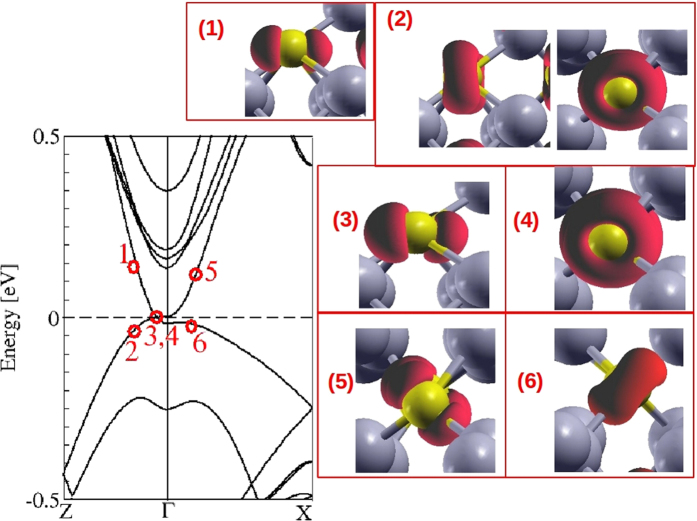
Atomic and orbital character of electronic states near a Dirac point. The corresponding eigenvalues are indicated in the band structure inset by the same numeral.

The existence of 3D cones at Dirac nodes **k**_±_ has been confirmed by other calculations^13^ and ARPES measurements^15^,^16^,^29^. The orbital character of the band states forming Dirac cones above and below the Fermi energy near the Dirac node **k**_±_ at the ΓZ line is illustrated in Fig. 9. By means of the orbital projection technique we find that all these states around the Dirac nodes possess an As *p* character. The topological character of the bct Dirac semimetal therefore gives rise to a completely different orbital character of the bands crossing near the Γ point in comparison to that of the artificial sc Cd_3_As_2_ crystal. Instead of Cd *s* here only As *p* states do contribute. Which *p* orbital contribute depends, however, on the direction of the **k** vector. This is demonstrated by their wave function squares plotted also in Fig. 9. They indeed indicate the dominance of As *p* states, mainly those pointed toward Cd vacancies. In contrast to the in-plane honeycomb arrangement of perpendicular C *p*_*z*_ orbitals in 2D graphene, in the topological Dirac semimetal Cd_3_As_2_ more or less all As atoms contribute. We trace this fact back to the appearance of Cd vacancies in all directions due to their helix arrangements. The orientation of the As *p* orbitals depends on the **k** value and on the energy above or below the Fermi energy. Along ΓZ the HOMO and HOMO − 1 states mainly possess *p*_*z*_ character, while *p*_*x/y*_ orbitals form the LUMO and LUMO + 1 states.

#### Optical spectra

**Figure 10:**
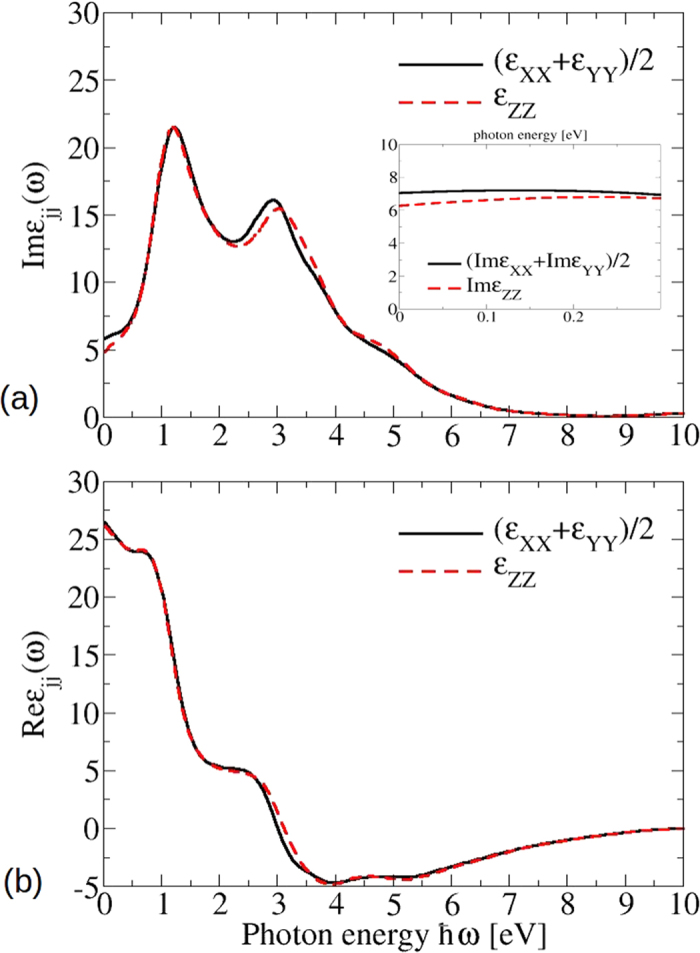
Real (**a**) and imaginary (**b**) part of the dielectric function for the bct phase. Inset: low energy limit for the imaginary part of the dielectric functions, calculated with a more dense **k**-point grid.

The optical spectra of bct Cd_3_As_2_, which result from the band structure in Fig. 5, are plotted in Fig. 10. More precisely, the real and imaginary parts of the dielectric function are displayed for light polarization parallel and perpendicular to the tetragonal axis. Above ~2 eV they show more or less the same lineshape as the corresponding spectrum for the artificial sc Cd_3_As_2_ presented in Fig. 4. The pronounced absorption peak near *ħω* = 2.6 eV in Fig. 10a still appears with almost the same intensity. However, the spectra for small photon energies *ħω* < 1.5 eV are totally different. The spectral weights are redistributed to guarantee the oscillator strength sum rule. An additional pronounced peak appears at *ħω* ≈ 1.2 eV, whereas the imaginary parts approach a small positive value for *ω* → 0. Both observations are consequences of the modified band structure in Fig. 5. The additional peak is due to van Hove singularities in the joint density of states at the lowest optical transition energies near *Z* and *N* points. The low-energy behavior is instead dominated by the non-conical 3D Dirac cones. In contrast to the linear-energy variation of the joint density of states for Dirac cones in 2D graphene-like systems^28^,^34^, in the 3D case linear bands give rise to a quadratic energy behavior of the joint density of states. Together with finite optical transition matrix elements in Table 1 and the *ω*^−2^ prefactor in expression (8) in the Methods section, finite imaginary parts 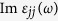 arise in the limit *ω* → 0.

In order to discuss more quantitatively the low-frequency behavior we first study the optical matrix elements, although only pseudo wave functions are available. Most important is that the momentum matrix elements at the Dirac nodes **k** = **k**_±_ are finite in Table 1, despite the vanishing optical transition energy. In the case of 2D graphene we have learnt^34^ that the matrix element squares averaged over the degenerate states (**k** ≈ **k**_±_)


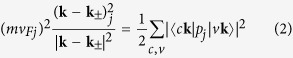


can be directly related to the Fermi velocity. With the values in Table 1 formula (2) leads to *v*_*Fx/y*_ = 0.78 × 10^5^ m/s and *v*_*Fz*_ = 0.73 × 10^5^ m/s. These values are of the order of magnitude of the geometric means of the Fermi velocities derived from Fig. 8, 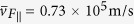 and 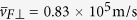. Their deviations in the case of the asymmetric 3D cones are not only a consequence of the use of pseudo wave functions, but also express the higher complexity of the ‘3D graphene’ with a varying orientation of the As *p* orbitals forming the linear bands (see Fig. 9).

The matrix elements (2) and the energy bands (1) allow the direct calculation of 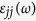 (see (8) in Methods section) in the limit *ω* → 0, where only two degenerate conduction (LUMO) and two degenerate valence (HOMO) states near the two Dirac points **k**_±_ contribute. Approximately the direction dependence of the momentum matrix elements is assumed to be equal to the wave vector as known from the Dirac physics for 2D materials^31^,^34^. Considering the twofold band degeneracy and the two Dirac nodes in the BZ, it can be analytically shown that for *ω* → 0 it holds


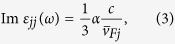


where the Sommerfeld finestructure constant *α* = *e*^2^/*ħc* and an effective Fermi velocity for the Cartesian direction *j*


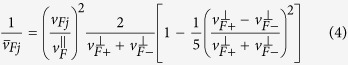


are applied. In the limit of isotropic Dirac cones and electron-hole symmetry 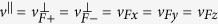 expression (4) agrees with literature findings^22^ (apart from a factor 2).

With the values 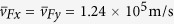 and 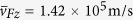 resulting from eq. 4 with the fit values in Fig. 8 one obtains the values 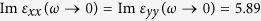 and 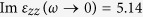. These values are in rough agreement with the results of the *ab initio* calculations performed using eq. 8 and displayed in Fig. 10. The *ab initio* values 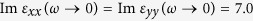 and 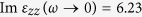 are however somewhat larger indicating smaller effective Fermi velocities, in agreement with the above discussion of linear and pseudolinear bands. The differences between the imaginary parts at zero frequency, characterizing the anisotropy of the crystal, 0.75 (analytic formula (3)) and 0.80 (*ab initio* calculations) are however close to each other. The small deviation is mainly due to the neglect of the **k** dispersion of the momentum matrix elements (2) away from the Dirac points.

Experimentally the optical reflectivity of [001] and [112]-oriented *n*-doped Cd_3_As_2_ crystals has been studied in a wide frequency range *ħω* = 0.006–2.8 eV^22^,^23^. Using the Kramers-Kronig relation also the real part of the optical conductivity (in cgs units)


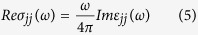


has been derived. It is isotropic within the (001) plane in agreement with the tetragonal symmetry. Because of relation (5) with (3) we predict for undoped samples


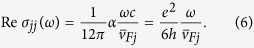


**Figure 11:**
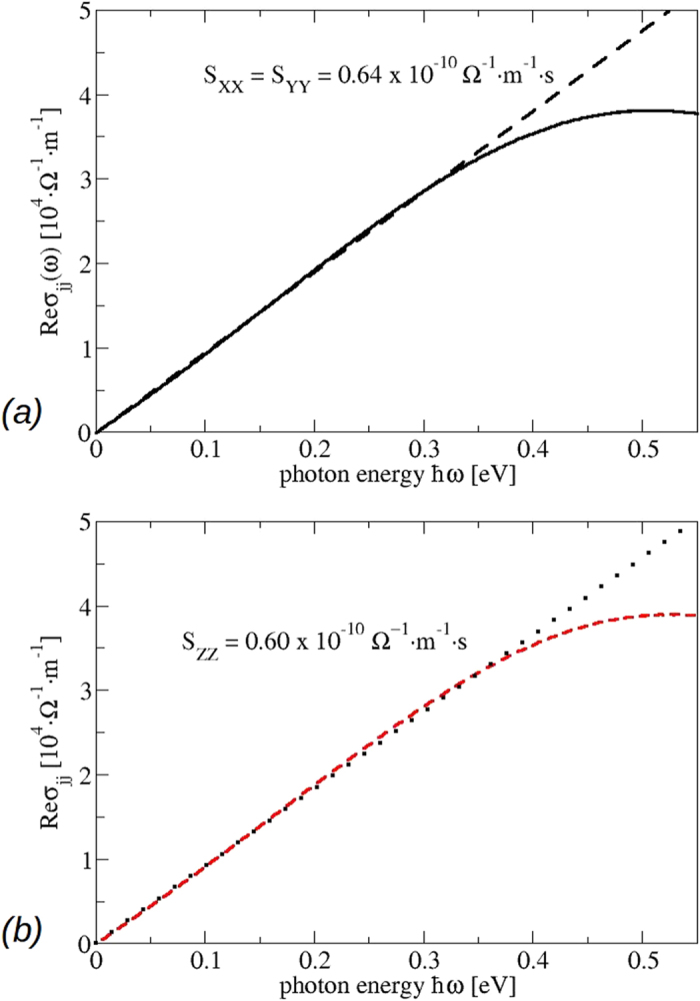
Real part of the optical conductivity for the bct phase. (**a**) Black: *xx* and *yy* component, (**b**) red: *zz* component. The slope parameters *S*_*jj*_ characterizing the constant imaginary parts of the dielectric tensor are also given.

The right-hand side of (6) is by a factor 2 smaller than the derivation in ref. 22 but agrees (apart from *ħ*^2^ as a misprint) with theoretical studies for systems with electron-hole symmetry and isotropic Dirac cones^35^. The reciprocal von Klitzing constant 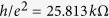 leads to the conductivity in SI units. The linear frequency dependence in (6) also appears in Fig. 11 for small frequencies *ħω* < 0.4 eV independent of the polarization direction. The absolute conductivity values reasonably agree with the measurements^22^,^23^. The slope coefficient


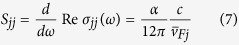


with values *S*_*xx*_ = *S*_*yy*_ = 0.52 × 10^−10^ Ω^−1^ *m*^−1^ *s* and *S*_*zz*_ = 0.45 × 10^−10^ Ω^−1^ *m*^−1^ *s* computed with the effective Fermi velocities 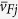 using eq. 4 agree with the *ab initio* calculations 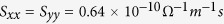 and *S*_*zz*_ = 0.60 × 10^−10^ Ω^−1^ *m*^−1^ *s* shown in Fig. 11. The lower slope parameter along the tetragonal axis is in qualitative agreement with recent optical measurements^23^. Most interesting is the linear behavior of the conductivities in the wide energy interval 0–0.3 eV. This is due to the pseudolinearity of the bands above and below the Dirac points, as discussed above. For very small frequencies (0.0–0.01 eV) a linearity with a different slope is expected, due to the linear bands at the Dirac points (see Fig. 8). To make it visible, one has to sample the BZ around the **k**_**±**_ with a more dense **k**-point grid. Moreover, the spectrum, in this low energy range, is modified by the Drude term in a real sample. The measured curve for Re*σ*_*xx*_(*ω*) shows an almost linear behavior in the energy range *ħω* = 0.16–0.74 eV^22^ with a slope parameter of about *S*_*xx*_ = 0.70 × 10^−10^ Ω^−1^ *m*^−1^ *s*, which is not too far from our computed value 0.64 × 10^−10^ Ω^−1^ *m*^−1^ *s*. For small frequencies the experimental spectrum shows several peaks. The power-law fit of the spectra in a wider energy range shows some deviations. The non-linear frequency dependence can be traced back to the sublinear **k** dispersion of the bands away from the Dirac nodes^35^.

A word of caution is needed, comparing calculated optical spectra with measured ones in the far-infrared region *ħω* smaller or equal 30 meV. In this spectral region our prediction of a linearly varying frequency dependence of the optical conductivity is modified by two effects: (i) Polar optical lattice vibrations will contribute to the optical absorption (see Fig. 1b in ref. 23). (ii) At finite (e.g. room) temperature the occupation of the Dirac cones near the Fermi energy will be slightly modified according to the Fermi function. The resulting free electrons and holes will give rise to Drude terms, which are hardly visible because of their small plasma frequencies. Both effects may slightly influence the limit *ω* → 0 and hence the slope parameter of the conductivity. Nevertheless,we can state that, despite of the strong anisotropy of the Dirac cones being visible in Fig. 7, the optical conductivity, as an integral quantity, exhibits linear frequency slopes. Those are certaintly dominated by non-conical Dirac fermions. On the other hand, in the higher frequency limit the slopes may be influenced by almost massless Kane-like fermions (see discussion in refs 23 and 36). However, the bands in Figs 5 and 7 clearly indicate that the interpretation in terms of massless Kane-like fermions is too simple. The complexity of the band structures around the Dirac points and Γ, in particular in the conduction band region along Γ*Z*, suggests that for photon energies *ħω* around 0.5 eV or larger optical transitions, in which no or only one Dirac cone is involved, play a role. The optical spectra ask for a more careful interpretation beyond Dirac fermions or even massless Kane-like fermions.

**Figure 12:**
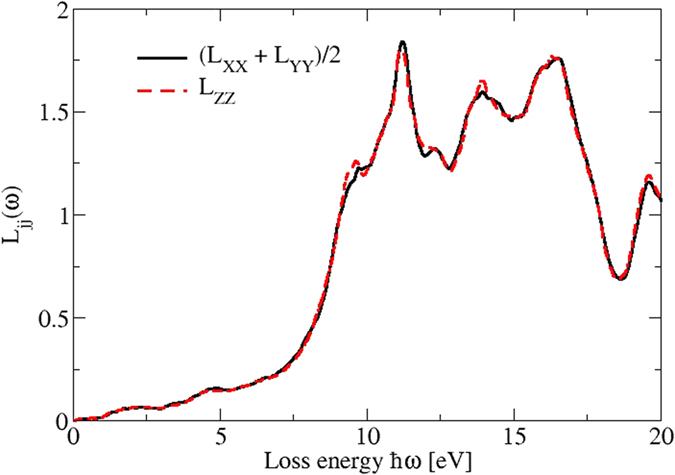
Energy loss spectra for vanishing momentum transfer but propagation along the Cartesian axes of the Cd_3_As_2_ 80-atom bct crystal. The different directions are displayed by colored lines.

For intermediate energies the fine structure of the energy loss spectra in Fig. 12 is due to the details of the band structure. However, the high-energy behavior of the loss functions is also influenced by collective plasmonic excitations of the valence electron with approximate plasma frequencies 
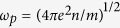
 and corresponding valence electron densities *n*. According to the band structure in Fig. 2 the valence electrons can be grouped into such due to As *p, s* and Cd *d* electrons. Since only the valence *s* and *p* electrons can be nearly treated as free electrons in the formula for the plasma frequency, two peaks in the loss spectrum near 10.6 eV (only *p* electrons) and 13.5 eV (*s* and *p* electrons) can be explained. Because of the stronger binding, the write effective mass of the Cd *d* electrons is larger than the free-electron mass. Consequently two other structures near 12.5 and 15.1 eV may be related to collective excitations where the *d* electrons partially contribute. The pronounced peak around *ħω* = 20 eV can be explained by contributions from the lowest As *s* and Cd5*d* bands into higher-lying conduction bands with *p* character.

## Summary and Conclusions

We have studied the most important electronic and optical properties of bct Cd_3_As_2_ crystals with 80 atoms in the unit cell by means of the *ab initio* density functional theory for calculations of the total energy and the electronic structure. The Kohn-Sham eigenvalues and eigenfunctions including the spin-orbit interaction have been used to predict band structures and the independent components of the frequency-dependent dielectric tensor. The results have been compared with recent ARPES and optical reflectivity measurements.

We have demonstrated that important electronic properties are already present in an artificial crystal consisting of 10-atom cubes. This holds especially for the tendency of an inverted band structure near the Γ point. However, the topology of the arrangement of the vacancies plays the most important role for the occurrence of Dirac points. In the inversion-symmetric bct geometry with large unit cells Cd_3_As_2_ appears as a Dirac semimetal with two Dirac nodes **k**_±_ at the tetragonal axis near the Γ point. The Fermi energy is pinned at the Dirac nodes, so that the Fermi surface only consists of two separate points. Therefore, bct Cd_3_As_2_ may be also interpreted as a multi-valley zero-gap semiconductor as graphene. For small energy variations we indeed found linear bands near **k**_±_, which however show a violation of the electron-hole symmetry and a strong asymmetry of the resulting Dirac cones in contrast to 2D graphene. The characteristic three Fermi velocities are of the order of magnitude (but smaller) as derived from ARPES experiments. The linear bands are formed by As *p* orbitals with varying orientation, underlining the interpretation of bct Cd_3_As_2_ as a 3D graphene. We suggest experiments with higher resolution to resolve the strong anisotropy of the Dirac cones along the tetragonal axis for small energies.

Despite the vanishing excitation energy the optical transition matrix elements at **k**_±_ are finite. Together with the band linearity and the vanishing gap we have derived constant but finite imaginary parts of the dielectric tensor. We demonstrate that the low-frequency value is governed by the Sommerfeld finestructure constant and an effective averaged Fermi velocity. We relate the measured dependence of the optical conductivity on the light polarization to the anisotropic Dirac cones for not too large photon energies. For energies in the range of about 0.5 eV the slopes of the optical conductivity are influenced by Kane-like fermions, also with linear band dispersions.

## Methods

In order to compute the structural, electronic and optical properties of Cd_3_As_2_ we apply the Density Functional Theory (DFT) as implemented in the code Quantum Espresso package^37^. Exchange and correlation (XC) are treated within the generalized gradient approximation (GGA) of Perdew, Burke and Ernzerhof (PBE)^38^. The electron-ion interaction is described by ultrasoft pseudopotentials for the *s* and *p* electrons but also for the low-lying Cd4*d* electrons^39^. Scalar relativistic corrections (mass and Darwin terms) and spin-orbit interaction (SOI) are taken into account. The inclusion of SOI has been done by first solving an all-electron radial Dirac-like Kohn-Sham equation for each atomic species and then taking the two upper components of the Dirac spinor electronic states in order to build the pseudopotential projectors^40^,^41^.

The wavefunctions are expanded into plane waves up to an energy cutoff of 80 (70) Ry for small (large) unit cells with 10 (80) atoms. The Brillouin Zone (BZ) is sampled by a uniform grid of 4 × 4 × 4 (2 × 2 × 2) Monkhorst-Pack **k**-points^42^ which are displaced in [111] direction by half a grid step. The atomic positions are determined by relaxing the atomic coordinates for given lattice constants until the Hellmann-Feynman forces are smaller than 1 meV/Å. Since quasiparticle corrections to the positions of the band extrema are vanishing^24^ around the Fermi level we restrict ourselves to the Kohn-Sham eigenvalues of the DFT. On an absolute energy scale there is a tendency for compensation of the blueshift of the spectra due to quasiparticle effects with their redshift due to excitonic effects^24^, hence they are neglected. Such effects may only slightly increase the wave-vector dispersion. Indeed, in the case of graphene and other 2D honeycomb material the Fermi velocities increase by about 15–20% due to quasiparticle effects^30^,^31^.

The frequency-dependent dielectric tensor 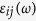 is calculated within the independent-particle approach^43^. The optical absorption is dominated by the imaginary part of its diagonal elements (*j* = *x, y, z*)^24^


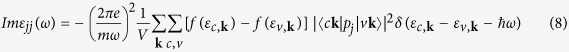


with *V* as the crystal volume. Here only interband transitions between occupied (|*v***k**〉) and empty (|*c***k**〉) Bloch states with 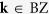 and corresponding eigenvalues 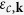 and 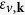 are taken into account. Their occupation is described by a smeared Fermi distribution *f *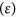. The real part of (8) follows via the Kramers-Kronig relation^24^. The strength of the optical transitions in (8) is governed by the matrix elements of the *j*^*th*^ component of the momentum operator **p**. For the simple cubic geometry we have used a mesh of 30 × 30 × 30 **k**-points, whereas a mesh of 5 × 5 × 5 **k** has been used for the bct phase. For the low energy limit of 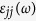 we have used a more dense mesh of **k**-points around the Dirac nodes. The dielectric function (8) is directly related to the optical conductivity tensor 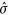
(*ω*) (see eq. 5). Therefore, the imaginary part in (8) not only allows the calculation of the real part via the Kramers-Kronig relation but also real and imaginary parts of the components of the conductivity tensor. In addition, by means of both quantities also the energy loss function 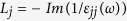 for vanishing momentum transfer can be derived.

## Additional Information

**How to cite this article:** Conte, A. M. *et al*. Electronic and optical properties of topological semimetal Cd_3_As_2_. *Sci. Rep.* **7**, 45500; doi: 10.1038/srep45500 (2017).

**Publisher's note:** Springer Nature remains neutral with regard to jurisdictional claims in published maps and institutional affiliations.

## Figures and Tables

**Table 1:** Optical matrix elements 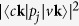 at a Dirac node **k** = **k**_±_ with a vanishing transition energy 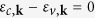 in atomic units 10^−2^ (*ħ/a*_*B*_)^2^.

*v*	*c*	*j* = *x*	*j* = *y*	*j* = *z*
HOMO	LUMO	5.263	3.224	4.396
HOMO	LUMO + 1	7.275	9.314	6.68
HOMO − 1	LUMO	7.275	9.314	6.668
HOMO − 1	LUMO + 1	5.263	3.224	4.396
Tot 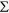		25.077	25.077	22.145

Two energetically degenerate valence (conduction) bands *v* = HOMO, HOMO − 1 (*c* = LUMO, LUMO + 1) are studied. The sums of all transitions are listed, too.
